# Characteristics of Dysregulated Proinflammatory Cytokines and Cognitive Dysfunction in Late-Life Depression and Amnestic Mild Cognitive Impairment

**DOI:** 10.3389/fimmu.2021.803633

**Published:** 2022-01-05

**Authors:** Jing Nie, Yuan Fang, Ying Chen, Aisikeer Aidina, Qi Qiu, Lu Zhao, Xiang Liu, Lin Sun, Yun Li, Chuwen Zhong, Yuan Li, Xia Li

**Affiliations:** ^1^ Shanghai Mental Health Center, Shanghai Jiaotong University, School of Medicine, Shanghai, China; ^2^ Department of Otolaryngology-Head and Neck Surgery, Shanghai Ninth People’s Hospital, Shanghai Jiao Tong University School of Medicine, Shanghai, China; ^3^ Ear Institute, Shanghai Jiao Tong University School of Medicine, Shanghai Key Laboratory of Translational Medicine on Ear and Nose Diseases (14DZ2260300), Shanghai, China

**Keywords:** late-life depression, amnestic mild cognitive impairment, neuroinflammation, cytokines, chemokines

## Abstract

**Background:**

Late-life depression (LLD) and amnestic mild cognitive impairment (aMCI) are two different diseases associated with a high risk of developing Alzheimer’s disease (AD). Both diseases are accompanied by dysregulation of inflammation. However, the differences and similarities of peripheral inflammatory parameters in these two diseases are not well understood.

**Methods:**

We used Luminex assays to measure 29 cytokines simultaneously in the plasma of two large cohorts of subjects at high risk for AD (23 LLD and 23 aMCI) and 23 normal controls (NCs) in the community. Demographics and lifestyle factors were also collected. Cognitive function was evaluated with the Chinese versions of the Montreal Cognitive Assessment (C-MoCA) and neuropsychological test battery (NTB).

**Results:**

We observed a remarkably increased level of IL-6 in the plasma and reduced levels of chemokines (CXCL11 and CCL13) in the LLD group compared with the aMCI group. The LLD group also showed lower levels of CXCL16 than the NC group. Furthermore, altered cytokine levels were associated with abnormal results in neuropsychological testing and Geriatric Depression Scale scores in both the LLD and aMCI groups. Notably, combinations of cytokines (IL-6 and CCL13) and two subitems of C-MoCA (orientation and short-term memory) generated the best area under the receiver operating characteristic curve (AUROC = 0.974).

**Conclusion:**

A novel model based on proinflammatory cytokines and brief screening tests performs with fair accuracy in the discrimination between LLD and aMCI. These findings will give clues to provide new therapeutic targets for interventions or markers for two diseases with similar predementia syndromes.

## Introduction

It is expected that by 2050, the number of people with dementia may exceed 131 million. Alzheimer’s disease (AD) is the most common type of dementia and is recognized by the WHO as a global public health priority (1). There are no effective medical treatments to cure the disease. However, the prevalence of dementia would be halved if its onset was delayed by 5 years (2). Therefore, the prodromal stage of AD is a great opportunity for effective treatment and postponement of disease onset.

Peripheral inflammatory activation potentially activates the immune system within the blood–brain barrier, playing a potential role in the pathogenesis of AD. Direct and bystander damage from inflammatory mechanisms is likely to significantly exacerbate the very pathogenic processes of AD along a continuum (3). Cytokines are typically produced by cells of the immune system upon activation and play key roles in the development and control of immune responses. Chemokines represent one of the largest subfamilies of cytokines and have been divided into several subgroups based on their chemical structures. CXC and CC are two major subfamilies, depending on whether the first two N-terminal cysteines have an amino acid between them (CXC) or are adjacent to each other (CC) (4). Although chemokines have been relatively neglected in investigations of the mechanisms of psychiatric disorders, recent evidence has begun to demonstrate an association between chemokines and neurobiological processes (5, 6). Dysfunctional cytokines have been associated with both neurodegenerative and psychiatric disorders, including AD, mild cognitive impairment, schizophrenia, depression, and bipolar disorder (7, 8). Increased inflammatory markers such as soluble tumor necrosis factor receptor 2 (sTNFR2), interleukin-6 (IL-6), and monocyte chemoattractant protein-1 (MCP-1) have been reported in patients with dementia compared with controls (9, 10). Although the exact effect of proinflammatory cytokines in AD remains unknown, increasing evidence suggests that inflammation is involved in the pathological process of AD and plays a crucial role in the early stages of disease when intervention may be most beneficial (11).

Late-life depression (LLD) and amnestic mild cognitive impairment (aMCI) subtypes are strongly associated with an increased risk for AD (12). Evidence from prospective studies has supported the notion that LLD is associated with a 2- to 5-fold increased risk of developing AD (13). Older adults with aMCI show typical impairment of episodic memory, which represents the most common symptom of the prodromal stage of AD (14). Compared with 3% of the population without mild cognitive impairment at the same age, approximately 39% of patients with mild cognitive impairment develop dementia over the subsequent 3 to 10 years (15). Although LLD and aMCI are considered to be distinct clinical entities, they share multiple common clinical features (16). Elderly individuals with major depressive disorder can present with significant cognitive impairment, decreased activity, primary motivational impairment, and fewer mood symptoms (17, 18). Cognitive dysfunction in depressed older adults typically consists of memory impairment, poor attention, and impaired executive function (19). Depression is also common in individuals with MCI, with a higher prevalence in aMCI than in non-aMCI (naMCI) and may confer a higher possibility of progression to dementia (20). Identifying individuals at risk for AD or depression will be critical, as clinical trials on prevention interventions or potential treatment may become available in the clinic.

Since neuroinflammation is thought to be involved in the pathogenesis of the disease at an early stage, it is also important to study inflammatory markers in the predementia stage. However, in previous inflammatory marker studies, elderly individuals with major depressive disorder were generally neglected. Therefore, in the current study, we aimed to characterize neuroinflammation in the elderly with aMCI (aMCI) and LLD compared with sex- and age-matched normal controls. Additionally, potential correlations between clinical parameters of proinflammatory cytokines and two diseases were analyzed. This study provides new diagnostic biomarkers targeting neuroinflammation for patients in the predementia stage.

## Materials and Methods

### Subjects and Study Procedure

**Figure 1:**
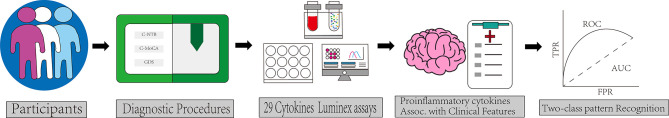
Study design and group comparisons. Participants from Shanghai, China, underwent cytokine level measurements, and 29 cytokines were selected for group comparisons. Receiver operating characteristic (ROC) analysis was adopted to evaluate the ability of cytokines to distinguish two-class patterns.

**Figure 1** describes the study design and analysis pipeline. Twenty-three participants diagnosed with aMCI and twenty-three normal controls were concurrently recruited from four different communities in Shanghai. The same number of patients with LLD was recruited according to the *Diagnostic and Statistical Manual of Mental Disorders*, Fifth Edition (DSM-V), by clinicians from an outpatient clinic (21).

All participants received clinical diagnoses established by three different geriatric psychiatrists using standardized assessment and review. Clinical diagnosis of aMCI was adapted from Petersen et al. (22), requiring evidence of a definite decline in memory (memory complaints corroborated by an informant and MoCA scores of >1.5 SD of age-appropriate norms or abnormal memory function for age) and without significant impact on daily living, with the severity of symptoms or consequent functional limitation not meeting the criteria for the diagnosis of dementia in DSM-V; a diagnosis of normal control was made if participants demonstrated no evidence of cognitive decline as compared with their baseline cognitive functions on clinical interview and assessment. Patients with LLD were diagnosed according to DSM-V for major depressive disorder with depression onset occurring over 60 years old. The exclusion criteria were as follows: a) dementia or suspected dementia based on clinicians’ judgment, b) other psychiatric disorders (except depression) or a substance-use disorder diagnosis, c) any kind of chronic infectious or any sign of peripheral inflammation, or d) any current, clinically significant cardiovascular and cerebrovascular disease.

### Clinical Data Collection

All participants were matched according to the mean age and sex. A detailed psychiatric, medical, social, and family history was obtained from each participant. All participants completed the following subtests: Chinese version of Montreal Cognitive Assessment (C-MoCA), Chinese version of neuropsychological test battery (C-NTB) including Wechsler’s Memory digit span (WMDS), Category Fluency Test (CFT), Controlled Word Association Test (COWAT), and Geriatric Depression Scale (GDS). This study was approved by the Institutional Review Board of the Shanghai Mental Health Center. Written informed consent was obtained from all participants or their representatives.

### Sample Collection and Preparation

Fasting blood samples of CRP were collected *via* peripheral venous access after participants fasted for more than 10 h. After centrifugation (2,500 rpm, 15 min, at 4°C), plasma was collected from EDTA tubes and then stored at −80°C until the assays were performed.

### Plasma Cytokine Assays

Using Luminex microbeads, we simultaneously measured the concentrations of 29 cytokines and chemokines (**Table 1**). The plasma samples were thawed at 4°C and centrifuged at 1,500 rpm to remove any aggregate protein that could potentially obstruct the measurement. The human Magnetic Luminex Performance Assay (R&D Systems, no. LXSAHM-33, USA) was used according to the manufacturer’s protocol. An X200 (Luminex, Austin, TX) was used to read the multiplex assay.

**Table 1:** The list of 29 human chemokines or cytokines tested.

CCL1/TCA-3	CCL2/MCP-1	CCL3/MIP-1α	CCL7/MCP-3	CCL8/MCP-2
CL11/Eotaxin	CCL13/MCP-4	CCL15/MIP-1δ	CCL17/TARC	CCL19/MIP-3β
CCL20/MIP-3α	CCL23/MPIF-1	CCL24/Eotaxin-2	CCL25/TECK	CCL27/CTACK
CXCL5/ENA-78	CXCL6/GCP-2	CXCL8/IL-8	CXCL9/MIG	CXCL11/I-TAC
CXCL16/SCYB16	IL-1β	MIP2	IFN-γ	VCAM-1
TNF-α	IL-2	IL-4	IL-6	

### Statistical Analysis

Statistical analysis was performed using SPSS Version 22 and GraphPad Prism software (version 6.0; GraphPad, Inc.). The results are reported as mean ± SD and numbers with percentages. Student’s t-test or Mann–Whitney U test for two groups and ANOVA or Kruskal–Wallis test for more than two groups were used to determine statistical significance. Pearson’s chi-squared test was used to compare categorical variables in the demographic data of the three groups. To examine associations between proinflammatory cytokines and cognitive assessment scores, analysis of partial correlation was used while adjusting for the demographic variables that differed statistically among the three groups.

We then evaluated the potential diagnostic and classification performance of combinations of inflammatory biomarkers and subitems of cognition testing using a logistic regression approach. In this analysis, all biomarkers used in any particular combination were entered as predictors, and the diagnostic group was entered as the dependent variable, controlling for age, sex, and education. After model fitting, receiver operating characteristic (ROC) analysis was used to evaluate the ability of the model to discriminate population with aMCI from LLD (23). The area under the ROC curve (AUROC) with a 95% CI for the parameters was used to measure its accuracy to differentiate patients with and without depression. Statistical significance was set at *p* < 0.05 on a two-tailed test.

## Results

### Demographic and Clinical Characteristics

Twenty-three subjects in each group (NC, aMCI, and LLD) took part in our study. There was no significant difference between any of the groups in terms of age or male-to-female ratio. Years of education was higher in the normal control group than in the other two groups. In terms of lifestyle factors, a lower proportion of patients with LLD performed regular exercise or drink tea. As expected, the control group had significantly higher mean C-MoCA scores than the other groups, and MDS, CFT, and COWAT scores were also lower in the aMCI and depression groups than in the control group. Scores of the GDS were significantly higher in the depression group than in the control and aMCI groups (**Table 2**).

**Table 2:** Comparison of demographic characteristics and cognitive function between patients with late-life depression (LLD), subjects with amnestic mild cognitive impairment (aMCI), and normal controls (NC).

	aMCI (n = 23)	LLD (n = 23)	NC (n = 23)	Statistics
**Clinical demographic data**				
Age	73.0 (4.5)	71.9 (5.7)	72.5 (0.8)	0.665
Years of education	13.0 (3.4)	11.2 (2.9)^#^	15.2 (2.2)^#&^	<0.01
Gender (male:female)	10:13	8:15	9:14	0.833
**Lifestyle (Y:N)**				
Smoking	5:18	3:20	3:20	0.649
Drinking	6:17	4:19	4:19	0.097
Tea drinking habits	13:10	5:18*^#^	12:11	0.024
Regular exercise	21:2	6:17*^#^	17:6	<0.01
**Global Cognitive scales**				
C-MoCA score	20.8 (2.6)	18.8 (6.1)	26.3 (2.1)^#&^	<0.01
WMDS score	12.1 (2.8)	13.4 (3.1)	16.6 (3.3)^#&^	<0.01
CFT score	6.7 (2.5)	5.5 (3.6)	10.0 (4.2)^#&^	<0.01
COWAT score	12.1 (3.4)	9.3 (3.5)^#^	14.8 (3.3)^#&^	<0.01
**Geriatric Depression Scale**	7.0 (3.9)	22.0 (4.6)*^#^	5.1 (2.7)	<0.01

All values, except sex, anti-inflammatory medication, and lifestyle, are expressed as mean ± SD.

aMCI, amnestic mild cognitive impairment; LLD, late-life depression; NC, normal controls; C-MoCA, Chinese version of Montreal Cognitive Assessment; WMDS, Wechsler Memory Digit Span; CFT, Category Fluency Test; COWAT, Controlled Word Association Test.

^*^Significantly different from controls (p < 0.05).

^#^Significantly different from aMCI group (p < 0.05).

^&^Significantly different from LLD group (p < 0.05).

### Cognitive Assessment

**Figure 2:**
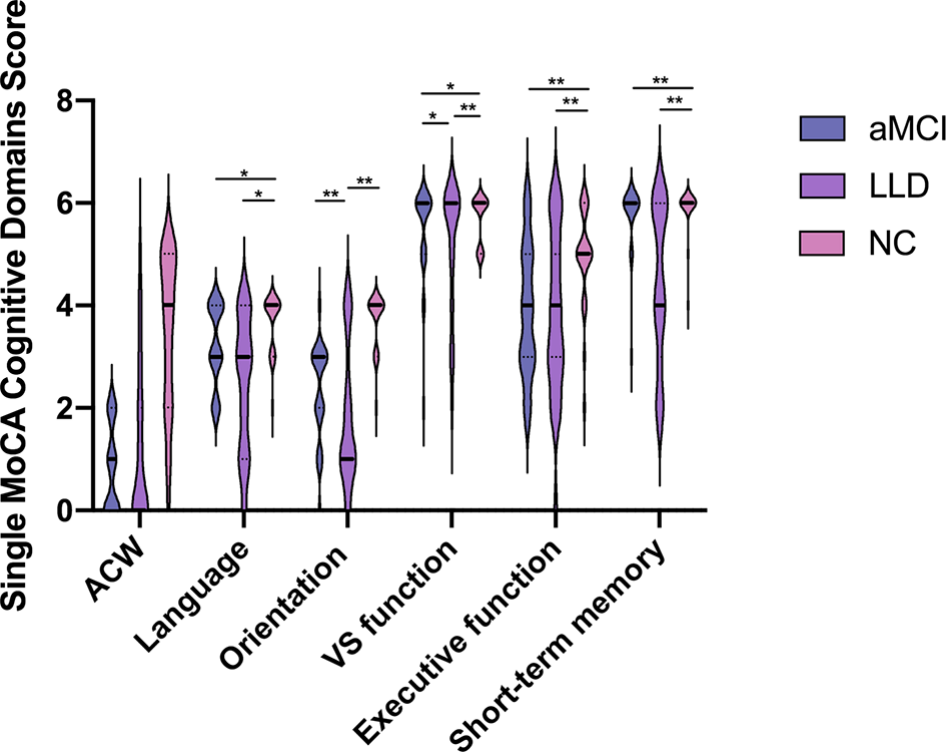
Scores for the 6 different neurocognitive domains tested. aMCI, amnestic mild cognitive impairment; LLD, late-life depression; NC, normal controls; ACW, attention, concentration, and working memory; vs. function, visual–spatial function. Data are expressed as mean ± SD. Statistical analysis was performed by the Kruskal–Wallis test; significance levels are indicated in the top portion of the figure (^*^ *p* < 0.05, ^**^ *p* < 0.01).

The comparison of the C-MoCA subitem scores among the three groups is shown in **Figure 2**.

All functional domains, with the exception of the attention, concentration, and working memory (ACW), were significantly changed between the disease and control groups. The LLD and aMCI groups scored significantly lower than the control group in language, short-term memory, and executive function. Surprisingly, LLD patients performed even worse than aMCI subjects in orientation and visual–spatial function tests [vs. function (aMCI: 3.2 ± 0.8 vs. LLD: 2.4 ± 1.3 vs. NC: 3.7 ± 0.6, *p* < 0.01), executive function (aMCI: 2.3 ± 0.9 vs. LLD: 1.8 ± 1.4 vs. NC: 3.7 ± 0.6, *p* < 0.01), ACW (aMCI: 5.4 ± 1.0 vs. LLD: 5.1 ± 1.3 vs. NC: 5.7 ± 0.5, *p* > 0.05), language (aMCI: 3.9 ± 1.3 vs. LLD: 3.8 ± 1.7 vs. NC: 4.9 ± 1.0, *p* < 0.05), orientation to time and place (aMCI: 5.7 ± 0.7 vs. LLD: 4.4 ± 1.5 vs. NC: 5.9 ± 0.5, *p* < 0.01), and short-term memory (aMCI: 0.8 ± 0.9 vs. LLD: 1.2 ± 1.6 vs. NC: 3.4 ± 1.6, *p* < 0.01)] (**Figure 2**).

### Inflammatory Protein Levels

**Figure 3:**
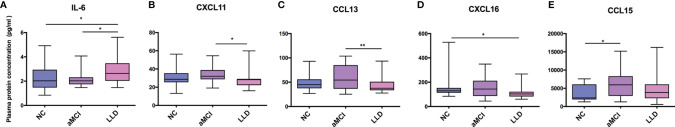
Chemokine and proinflammatory cytokine concentrations among patients with late-life depression and amnestic mild cognitive impairment and normal controls with standard error of the mean. **(A)** IL-6 among LLD, aMCI, and control group. **(B)** CXCL11 among LLD, aMCI, and control group. **(C)** CCL13 among LLD, aMCI, and control group. **(D)** CXCL16 among LLD, aMCI, and control group. **(E)** CCL15 among LLD, aMCI, and control group. aMCI, amnestic mild cognitive impairment; LLD, late-life depression; NC, normal controls; IL-6, interleukin-6; CXCL11, C-X-C chemokine ligand 11; CXCL16, C-X-C chemokine ligand 16; CCL2, C-C chemokine ligand 2; CCL13, C-C chemokine ligand 13; CCL15, C-C chemokine ligand 15. Significance levels are indicated in the top portion of the figure (^*^ *p* < 0.05, ^**^ *p* < 0.01).

**Figures 3A–E** show the protein concentrations of proinflammatory cytokines and chemokines. In total, patients in the LLD group had significantly higher levels of IL-6 (aMCI: 2.2 ± 0.6 vs. LLD: 2.9 ± 1.0 vs. NC: 2.3 ± 1.1, *p* < 0.05 **Figure 3A**) than the control or aMCI subjects. LLD patients showed lower levels of CXCL16 (127.8 ± 57.6 vs. 170.1 ± 122.1, *p* < 0.05 **Figure 3D**) than participants in the control group. Furthermore, the aMCI group showed a higher level of CCL15 (6,048.0 ± 3,608.5 vs. 3,713.7 ± 2,200.6, *p* < 0.01, **Figure 3E**) than subjects in the control group. Compared with depressed patients, the aMCI group had higher levels of CXCL11 (33.9 ± 8.3 vs. 28.3 ± 9.4, *p* < 0.05, **Figure 3B**) and CCL13 (60.1 ± 23.9 vs. 42.8 ± 14.2, *p* < 0.01, **Figure 3C**) in plasma. Meanwhile, no significant differences were noticed in plasma concentrations of other chemokines and cytokines among the three groups (**Table 3**).

**Table 3:** Level of inflammatory protein concentration in controls and patients.

	aMCI (n = 23)	LLD (n = 23)	NC (n = 23)	*P*
TNF-α (pg/ml)	5.2 (1.8)	8.7 (16.8)	5.5 (0.9)	0.280
CCL1 (pg/ml)	1.7 (0.9)	1.6 (0.8)	1.4 (0.5)	0.578
CXCL8 (pg/ml)	3.2 (2.2)	2.3 (1.7)	3.5 (2.8)	0.361
CCL7 (pg/ml)	20.3 (16.9)	13.6 (13.1)	17.7 (15.8)	0.282
MIP2 (pg/ml)	194.8 (140.4)	180.6 (260.8)	268.9 (259.4)	0.249
IL-1β (pg/ml)	3.7 (1.7)	4.5 (2.7)	3.3 (2.5)	0.175
IFN-γ (pg/ml)	26.6 (10.4)	24.9 (6.0)	24.2 (6.4)	0.741
CCL20 (pg/ml)	60.4 (29.5)	84.1 (117.1)	64.3 (54.6)	0.509
CCL24 (pg/ml)	550.5 (224.8)	446.4 (260.2)	553.1 (292.4)	0.050
CCL3 (pg/ml)	133.8 (32.5)	127.4 (41.5)	130.3 (49.4)	0.955
CCL8 (pg/ml)	279.5 (16.7)	278.4 (13.1)	275.7 (14.9)	0.557
IL-4 (pg/ml)	29.2 (6.5)	28.3 (5.0)	27.5 (5.9)	0.663
CCL19 (pg/ml)	115.9 (38.8)	158.4 (143.9)	114.4 (64.6)	0.668
IL-2 (pg/ml)	4.0 (1.9)	3.3 (2.5)	3.6 (2.2)	0.118
CXCL9 (pg/ml)	588.1 (80.7)	611.6 (81.4)	613.2 (89.3)	0.705
CCL23 (pg/ml)	421.5 (150.6)	454.6 (216.4)	458.5 (200.2)	0.784
VCAM-1 (ng/ml)	1,210.8 (452.7)	1,342.2 (594.8)	1,138.4 (599.4)	0.324
CCL27 (pg/ml)	508.8 (146.1)	488.2 (138.0)	508.5 (143.2)	0.880
CCL11 (pg/ml)	85.4 (31.5)	73.7 (17.4)	76.5 (42.2)	0.302
CXCL5 (pg/ml)	437.7 (308.0)	339.4 (223.2)	605.4 (481.5)	0.209
CXCL6 (pg/ml)	164.8 (90.2)	121.6 (62.7)	221.0 (191.4)	0.179
CCL25 (pg/ml)	172.9 (78.6)	145.2 (61.9)	205.2 (130.6)	0.258
CCL2 (pg/ml)	216.3 (117.8)	153.2 (72.5)	186.2 (98.6)	0.118
CCL17 (pg/ml)	171.0 (120.5)	115.5 (46.7)	151.1 (95.9)	0.181

All values are expressed as mean ± SD.

aMCI, amnestic mild cognitive impairment; LLD, late-life depression; NC, normal controls; TNF-α, tumor necrosis factor-alpha; IL-1β, interleukin-1-beta; (MIP)-2, macrophage inflammatory protein-2; IFN-γ, interferon-γ-inducing factor; IL-2, interleukin-2; IL-4, interleukin-4; VCAM-1, vascular cell adhesion molecule-1; CCL1, C-C chemokine ligand 1; CCL2, C-C chemokine ligand 2; CCL3, C-C chemokine ligand 3; CCL7, C-C chemokine ligand 7; CCL8, C-C chemokine ligand 8; CCL20, C-C chemokine ligand 20; CCL24, C-C chemokine ligand 24; CCL11, C-C chemokine ligand 11; CCL17, C-C chemokine ligand 17; CCL19, C-C chemokine ligand 19; CCL23, C-C chemokine ligand 23; CCL25, C-C chemokine ligand 25; CCL27, C-C chemokine ligand 27; CXCL5, C-X-C chemokine ligand 5; CXCL5, C-X-C chemokine ligand 6; CXCL8, C-X-C chemokine ligand 8; CXCL9, C-X-C chemokine ligand 9.

### Correlations of Peripheral Inflammatory Marker With Cognition and Depression Symptoms

**Figure 4:**
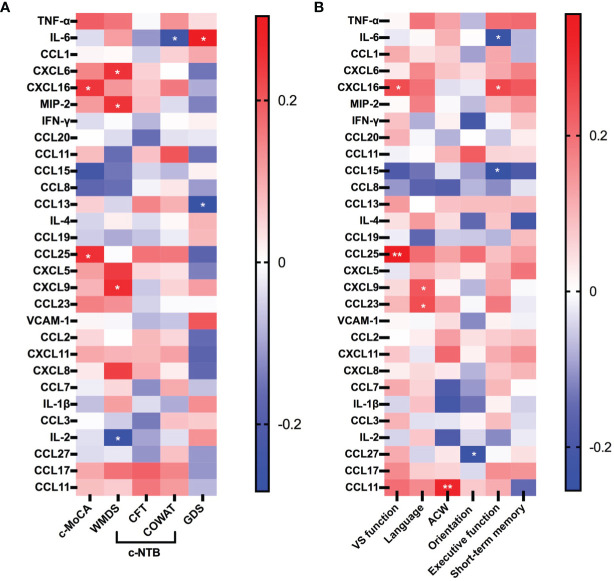
Heatmap showing correlations between the concentrations of cytokines and chemokines with clinical characteristics of LLD and aMCI patients. **(A)** Association among proinflammatory cytokines, chemokines, and clinical scores reflecting the severity of cognition and depression. **(B)** Association among proinflammatory cytokines, chemokines, and subdomains of cognitive scores. The six neurocognitive domains shown in **Figure 3B** are the subdomains of the MoCA; red color indicates a positive correlation, and blue indicates a negative correlation; *p*-values are presented, ^*^ *p* < 0.05, ^**^ *p* < 0.01. C-MoCA, Chinese version of Montreal Cognitive Assessment; WMDS, Wechsler Memory digit span; CFT, Category Fluency Test; COWAT, Controlled Word Association Test; GDS, Geriatric Depression Scale; ACW, attention, concentration, and working memory; vs. function, visual–spatial function. Six CXC chemokine ligands (CXCL5, CXCL6, CXCL8, CXCL9, CXCL11, and CXCL16) and 15 CC chemokine ligands (CCL1, CCL2, CCL3, CCL7, CCL8, CCL11, CCL13, CCL15, CCL17, CCL19, CCL20, CCL23, CCL24, CCL25, and CCL27). MIP2, microphage inflammatory protein-2; IL-2ha, interleukin-2; IL-4, interleukin-4; IL-6, interleukin-6; VCAM-1, vascular cell adhesion molecule-1; TNF-α, tumor necrosis factor-alpha; IL-1β, interleukin-1-beta; IFN-γ, interferon-γ-inducing factor.

Among LLD and aMCI patients, lower levels of CXCL16 (*r* = 0.247, *p* = 0.041) and CCL25 (*r* = 0.255, *p* = 0.035) were associated with worse C-MoCA summary scores, while controlling for age, sex, and education (**Figure 4A**). For further analysis, we explored the correlation between neuroinflammation and the subdomains of the MoCA. Levels of CXCL16 (*r* = 0.254, *p* = 0.035) and CCL25 (*r* = 0.356, *p* < 0.01) were also positively correlated with visual–spatial function. Levels of CXCL9 (*r* = 0.051, *p* = 0.048) and CCL23 (*r* = 0.101, *p* = 0.036) were positively associated with language function scores. The level of CCL11 was positively associated with ACW scores (*r* = 0.320, *p* < 0.01). A lower CCL27 concentration was found to be associated with higher orientation scores (*r* = −0.239, *p* = 0.048). The levels of CCL15 (*r* = −0.256, *p* = 0.034) and IL-6 (*r* = −0.243, *p* = 0.044) were found to be negatively associated with executive function scores, while the level of CXCL16 (*r* = 0.261, *p* = 0.030) was positively associated with executive function scores (**Figure 4B**).

A higher level of IL-6 was associated with lower COWAT scores (*r* = −0.251, *p* = 0.037) and increased GDS scores (*r* = 0.304, *p* = 0.011). CCL13 was also negatively associated with GDS scores (*r* = −0.283, *p* = 0.018), reflecting the severity of depression. Levels of CXCL6 (*r* = 0.250, *p* = 0.038), CXCL9 (*r* = 0.261, *p* = 0.030), and MIP2 (*r* = 0.251, *p* = 0.038) were positively associated with WMDS scores. Conversely, the level of IL-2 was negatively associated with WMDS scores (*r* = −0.265, *p* = 0.028) (**Figure 4A**).

### Combined Scale Items and Cytokines Across the Diagnostic Spectrum (Late-Life Depression/Amnestic Mild Cognitive Impairment)

**Figure 5:**
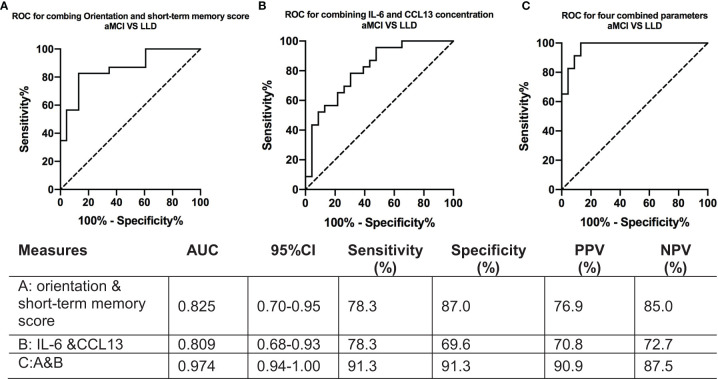
ROC curves for IL-6 and CCL13 concentrations between patients with late-life depression and amnestic mild cognitive impairment. **(A)** Receiver operating characteristic curve for combining orientation and short-term memory scores to discriminate between the aMCI and LLD groups. **(B)** Receiver operating characteristic curve for combining IL-6 and CCL13 to discriminate between the aMCI and LLD groups. **(C)** Receiver operating characteristic curve for combining the four parameters above to discriminate between the aMCI and LLD groups. AUROC, area under the receiver operating characteristic curve; aMCI, amnestic mild cognitive impairment; LLD, late-life depression; IL-6, interleukin-6; CCL13, C-C chemokine ligand 13. The AUROC curves result from a logistic regression model adjusted for age, sex, and education. ROC, receiver operating characteristic.

The results of the ROC analyses, which assessed the cognitive assessment score and cytokine concentrations in discriminating between the LLD and aMCI groups, are presented in **Figure 5**.

The orientation and short-term memory testing combination distinguished LLD patients from aMCI groups with an AUROC of 0.83 (95% CI, 0.70–0.95) (**Figure 5A**). The combination [IL-6+CCL13] also showed significant value for differentiating between groups, with an AUROC of 0.81 (95% CI, 0.68–0.93) (**Figure 5B**). Subitems of the C-MoCA [orientation + short-term memory] and [IL-6+CCL13] combination discriminated LLD patients from aMCI with an AUROC of 0.97 (95% CI, 0.94–1.00) (**Figure 5C**).

## Discussion

To our knowledge, this is the first study scrutinizing the diagnostic contribution of a peripheral inflammatory biomarker panel—IL-6 and CCL13, both as single markers and in combination with neuropsychological testing—in the biomarker-guided differential diagnosis of LLD and aMCI. We also found that several cytokines in patients with aMCI and LLD were markedly different, and the altered chemokines were partly correlated with the clinical parameters. The results of this study will provide new leads to further understanding the etiology of the preclinical stage of dementia and improve our ability to identify high-risk individuals in the clinical setting.

### Comparing Peripheral Inflammatory Markers in Late-Life Depression Versus Amnestic Mild Cognitive Impairment

Multiplex analytical technologies are crucial to the complex task of deciphering disease-specific biomarker patterns, as they provide opportunities for an all-inclusive approach, resolving the problem that different ELISA kits do not give identical absolute values of the same analyses. In our study, using a Luminex assay, a reliable multiplex analytical technology, we were able to measure multiple different cytokines simultaneously in a single run of the assay with small sample-size requirements (24). Simultaneous measurement of multiple cytokines in peripheral blood samples reveals objective changes in multiple cytokines within one experiment, which provides a more inclusive and comprehensive depiction of the inflammatory process in the preclinical stage of dementia.

IL-6 can be produced by a variety of cells, such as immune-mediated cells, endothelial cells, and fibroblasts, which mediate immune cell expansion in response to infections and tissue injuries (25). The cytokine IL-6 has been extensively investigated in many preclinical and clinical studies as a proinflammatory cytokine that can accelerate ongoing neurodegenerative processes in AD. Previous research found that plasma circulating IL-6 may be a useful indication for future cognitive function (26), and increased IL-6 levels were reported in LLD patients in peripheral blood (27). Leung showed that elevated IL-6 in AD patients versus controls negatively correlated with Mini-Mental State Exam (MMSE) scores (28). We found similar results in our study. In our study, we found that plasma IL-6 levels were significantly increased in patients with LLD and positively associated with GDS scores. Interestingly, we did not find significant associations between IL-6 concentration and the total C-MoCA score; however, an increase in IL-6 was associated with better verbal fluency scores.

CCL13/MCP-4 is a CC family chemokine that can induce crucial immunomodulatory responses through its effects on epithelial, muscular, and endothelial cells (29). CXCL11 is a ligand of CXCR3 and has a proinflammatory effect (30). In this study, we found significantly lower levels of plasma CCL13 and CXCL11 in LLD patients than in aMCI patients. In line with our finding, people with suicidal behavior reported a similar decrease in the levels of chemokines (e.g., IL-10; CCL1, CCL8, CCL13, CCL15, CCL17, CCL19, CCL20, and CXCL11) (31). The suicide rates in the elderly are higher than those in younger adults and are more closely associated with depressive symptoms (32). We also found that the level of CCL13 was negatively associated with GDS scores, reflecting the severity of depression. Low levels of this cytokine may indicate more severe depressive symptoms and a higher risk of actually committing suicide. Longitudinal follow-up of these participants is underway to confirm this hypothesis.

The demographic characteristics in the LLD group were likely associated with a reduction in some chemokines. Personal characteristics and a series of complex factors (cultural, poor physical health, and social isolation) affect whether a person, group, or community develops a late-onset major depressive disorder. In this study, the results showed that 26.1% of the elderly led a sedentary lifestyle, while 91.3% of mild cognitive impairment subjects performed regular physical exercises. Depressive symptoms are associated with decreased physical performance in older adults in both cross-sectional and longitudinal epidemiological studies (33, 34). However, the relationship between an increasing proportion of physical activity and depressive symptoms may be bidirectional. On the one hand, depression may lead to decreased levels of activity due to low motivation and energy. On the other hand, decreased physical activity could be a risk factor for depression. Physical activity is known to increase the secretion of multiple hormones with antidepressant properties: physical activities can temporarily change norepinephrine activity in the central system, decrease the activity of the hypothalamopituitary–adrenocortical axis, and increase the secretion of beta-endorphins (35, 36). To some extent, lower levels of chemokines may also be explained by the finding that patients with LLD tend to have a sedentary lifestyle and lack stimulation in life.

Multiple directions of regulation of cytokines and inflammation associated with proteins of AD and MCI patients have been described in different studies. CCL15 is classified as a macrophage inflammatory protein and has a proinflammatory effect (37). In this study, we found that plasma CCL15 was increased in aMCI subjects. It was negatively associated with executive function. Significant elevations in plasma CCL15 levels in AD have been reported by others; however, the data showed no changes in CCL15 in mild cognitive impairment (38). There are also conflicting data showing lower CCL15 levels in AD (39). Such results may be related to the severity of the disease and the heterogeneity of the MCI population. In previous studies, elevated peripheral vascular cell adhesion molecule-1, tumor necrosis factor (TNF)-α, IL-2, IL-6, IL-18, and interferon-γ were found in patients with mild AD, suggesting that cytokine signaling might play a role in the intermediate stages of dementia (40). The levels of CCL13, CXCL16, and CXCL11 were significantly lower in the LLD group than in the aMCI and NC groups in the present study. The results may indicate to some extent that a potential correlation between cytokine concentrations and the severity of cognitive dysfunction may not be apparent but may be more strongly correlated with mood in the early stages of the disease. These results suggest a different pattern of inflammatory dysregulation in LLD from aMCI, although they share similar clinical features. The findings herein raise the plausible possibility of LLD-induced immunosuppression and that the effects on cognition and mood are potentially mediated more significantly by IL-6 than other markers.

### Montreal Cognitive Assessment as a Clinical Marker in Combination With Peripheral Inflammatory Markers

Our data show that the combination of two cytokines and subitems of the MoCA enables satisfactory differentiation between LLD patients and mild cognitive impairment subjects. Notably, neither MoCA nor biomarkers (CCL13 and IL-6) alone were sufficient. Our discriminant analysis based on orientation, short-term memory, IL-6, and CCL13 yielded a high sensitivity of 91.3% for the identification of LLD patients. Diagnostic heterogeneity remains a major obstacle to understanding and treating patients with psychiatric disorders. The lack of strictly defined physiological targets limits advances in precision medicine for determining optimal treatments or developing new interventions. Sometimes, elderly individuals with depression accompanied by cognitive impairment are diagnosed with MCI by the clinician, whereas laboratory markers have the potential to identify AD-associated alterations at a very early stage. Rigorous identification of MCI in geriatric major depression treatment trials will help to advance the effectiveness of treatments (41, 42).

The C-MoCA is one of the most widely used cognitive assessments in China, and it has shown good sensitivity and acceptable specificity in differentiating MCI from older adults with normal cognition (43, 44). In our study, attention to the performance of single cognitive items, rather than only focusing on the total score of the C-MoCA, has the potential to improve our understanding of the earliest cognitive alterations in preclinical AD.

Treating AD can be challenging because pathological changes precede the diagnosis of clinical dementia for many years. Due to the lack of pharmacological treatments to slow the progression of dementia, therapies should be developed to target people at risk of dementia. Thus, the distinguished cytokine combined with two selected cognition tests will facilitate detection of people with cognitive impairment, allowing for further investigation of the proinflammatory cytokine effects on the preclinical AD and neurodegeneration diseases and adoption of individual preventive and therapeutic measures. Further work will need to evaluate the potential role of the model in the prediction of progression from the prodromal stage of dementia to the dementia stage.

The following limitations of our study need to be discussed. It is undeniable that the cross-sectional study design and small sample size limit cause–effect conclusions regarding the relationship between inflammation and LLD. The diagnosis of MCI was made on a clinical basis according to Petersen’s criteria. However, the revised NINCDS-ADRDA criteria have been reported to be reasonably accurate and are widely used in population-based studies (45). Here, recruited individuals refused to perform lumbar puncture and cerebrospinal fluid testing, which is an invasive screening method. PET imaging is expensive and difficult to accept in the elderly. All individuals were recruited from our cohort who had been diagnosed by three different geriatric psychiatrists. Given the aforementioned limitations, further studies are required to increase the statistical power by collecting larger, multisite cohorts and for detecting biomarkers of AD pathology, not only in predementia individuals but also across the AD diagnostic spectrum.

## Conclusions

The results described the plasma cytokine profile in LLD patients in comparison with aMCI patients and controls, identifying neurocognitive and clinical associations with peripheral inflammatory markers. The combination of levels of cytokines and subitems of neuropsychological testing may provide a cost-effective, simple method for differentiating between LLD and aMCI in individuals. These results suggest the involvement of different immunological mechanisms in two diseases with similar predementia syndromes, showing that further research on cytokines may inspire the development of approaches for preclinical dementia diagnosis and treatment.

## Data Availability Statement

The original contributions presented in the study are included in the article/supplementary material. Further inquiries can be directed to the corresponding authors.

## Ethics Statement

The studies involving human participants were reviewed and approved by Shanghai Mental Health Center ethical standards committee on human experimentation. The patients/participants provided their written informed consent to participate in this study.

## Author Contributions

JN, YF, and YC performed the statistical analysis and drafted the main manuscript text. AA, QQ, LZ, XLiu, LS, YunL, and CZ performed the experiments and acquired the data. YuaL and XLi were involved in the study conception, participated in the design and coordination, and helped to draft the manuscript. All of the authors helped to draft the manuscript and gave critical comments. All of the authors are acknowledged.

## Funding

This study was supported by grants from the National Natural Science Foundation of China National Natural Science Foundation of China (No. 81671402 & 81901170), National Key R&D Program of China (2017YFC1310500 and 2020YFC2005200), Clinical Research Plan of SHDC (SHDC2020CR1044B), and the Talent Development Program of Shanghai Mental Health Centre (2020-FX-04).

## Conflict of Interest

The authors declare that the research was conducted in the absence of any commercial or financial relationships that could be construed as a potential conflict of interest.

## Publisher’s Note

All claims expressed in this article are solely those of the authors and do not necessarily represent those of their affiliated organizations, or those of the publisher, the editors and the reviewers. Any product that may be evaluated in this article, or claim that may be made by its manufacturer, is not guaranteed or endorsed by the publisher.
